# Molecular Characterization of Mosaicism for a Small Supernumerary Marker Chromosome Derived from Chromosome Y in an Infertile Male with Apparently Normal Phenotype: A Case Report and Literature Review

**DOI:** 10.1155/2019/9398275

**Published:** 2019-11-19

**Authors:** Na An, Yang Yu, Qi Xi, Fagui Yue, Ruizhi Liu, Shibo Li, Ruixue Wang

**Affiliations:** ^1^Center for Reproductive Medicine, Center for Prenatal Diagnosis, First Hospital, Jilin University, Changchun 130021, China; ^2^Jilin Engineering Research Center for Reproductive Medicine and Genetics, Jilin University, Changchun 130021, China; ^3^Department of Pediatrics, University of Oklahoma Health Sciences Center, Oklahoma City, OK 73104, USA

## Abstract

Small supernumerary marker chromosomes (sSMCs), equal in size or smaller than chromosome 20 of the same metaphase, can hardly be identified through traditional banding technique. They are usually associated with intelligent disability, growth retardation, and infertility, but the genotype-phenotype correlations are still complicated for their complex origins and constitutions. Herein, we identified a 26-year-old Chinese infertile male who carried a mosaic sSMC and was diagnosed as severe oligospermia. The G-banding analysis initially described his karyotype as mos 47, XY, +mar[32]/46, XY[18]. The chromosomal microarray analysis results showed a 25.5 Mb gain in Yp11.31q11.23 and a 0.15 Mb loss in Yq12. Two *SRY* signals were discovered in the “seemingly” normal chromosome Y in both cell lines using *SRY* probe: one normal *SRY* was located on the distal tip of the short arm of chromosome Y while the other *SRY* was located on the terminal of long arm in the same chromosome Y. The sSMC(Y) was finally identified as der(Y) (pter ⟶ q11.23) (*SRY-*). To our knowledge, the chromosomal Y anomalies, *SRY* gene translocated from der(Y) (pter ⟶ q11.23) to qter of normal chromosome Y, were not reported before. Our findings indicated that the mosaic presence of sSMC(Y) may be the main cause of severe oligospermia although no other apparent abnormalities were observed in the proband. Further research on association between sSMC(Y) and spermatogenesis impairment should be investigated. It is recommended measures of traditional and molecular cytogenetic analysis should be taken to determine the origins and constitutions of sSMC so as to offer more appropriate genetic counseling for the infertile sSMC carriers.

## 1. Introduction

Small supernumerary marker chromosomes (sSMCs) are defined as structurally abnormal chromosomes, which cannot be characterized clearly by cytogenetic G-banding and require molecular approaches for definitive characterization. They are equal in size or smaller than chromosome 20 of the same metaphase spread and belong to a heterogeneous group. The incidence rate in general population was about 0.3–0.5/1000 [1]. Approximately 77% of sSMC are *de novo* and 23% were parentally inherited: maternal (16%) and paternal inheritance (7%) [2]. Approximately 1/3 sSMC cases are associated with specific clinic symptoms, for example, the i(18), der(22), i(12p) (Pallister Killian syndrome), and inv dup(22) (cat-eye) syndromes, while 2/3 sSMC cases have not been correlated with clinical syndromes [3]. The most common sSMC are derived from chromosome 15, accounting for 30∼50% [4]. Currently, it is difficult to define the phenotype-karyotype correlation of sSMC for their complex origins and genetic materials (euchromatin or heterochromatin) [5].

sSMCs, as a specific genetic imbalance, are usually discovered in patients with mental retardation, infertile couples, and prenatal cases [6]. The frequency of sSMC in individuals presenting infertility is higher than in the general population (0.125 vs. 0.043%). And, the incident rate is also varied between male (0.165%) and female infertility (0.022%) [7]. Male fertility could be reduced due to the existence of sSMC without additional clinical sSMC-related symptoms. The sSMC resulting from acrocentric chromosome would more easily lead to infertility [8]. It was estimated that 72% of sSMCs detected in association with infertility are derived from acrocentric chromosomes [6]. However, reports on sSMC(Y) associated with male infertility are limited [9].

Here, we present an infertile male with mosaic sSMC (*SRY*-) derived from chromosome Y through cytogenetic and molecular genetic analyses and analyze the reason for his infertility at the same time.

## 2. Materials and Methods

### 2.1. Patient

A 26-year-old Chinese infertile male was referred to our center for infertility consultation because of regular unprotected coitus and no pregnancy. His height was 170 cm and weight was 80 kg. He had normal mental development. Physical examination revealed normal penis and pubic hair. The volume for left and the right testicular was separately 12 mL. No other physical abnormalities were observed. Reproductive hormone levels were as follows: LH (luteinizing hormone): 1.70 mIU/ml (1.7∼8.5 mIU/ml), FSH (follicle-stimulating hormone): 3.70 mIU/ml (1.5∼12.4 mIU/ml), E2 (estradiol) 26.54 pg/ml (28∼248 pg/ml), and T (testosterone): 3.80 nmol/l (9.9∼27.8 nmol/l), PRL (prolactin): 291.00 uIU/ml (86∼258 uIU/ml). Semen examination was performed according to the World Health Organization guidelines (WHO 5th), and he was diagnosed as severe oligospermia. Our study protocol was approved by the Ethics Committee of the First Hospital of Jilin University (No. 2015-276), and the informed written consents were obtained from the patient and his father for publication of this case report and accompanying images.

### 2.2. Cytogenetic Analysis

Cytogenetic studies were performed on metaphases collected from cultured peripheral blood cells. Chromosome preparations were obtained according to G-banding techniques at 300–400 banding resolution. We analyzed fifty metaphases were for the proband and his father. The ISCN 2016 nomenclature was used to describe the karyotype [10].

### 2.3. Chromosomal Microarray Analysis (CMA)

Genomic DNA was isolated from 5 mL peripheral blood of the patient. Then, the procedures are conducted through CytoScan 750K array (Affymetrix, Santa Clara, CA, USA). The procedure included genomic DNA extraction, digestion and ligation, PCR amplification, PCR product purification, quantification and fragmentation, labeling, array hybridization, washing, and scanning. Thresholds for genome-wide screening were set at ≥200 kb for gains, ≥100 kb for losses. The detected copy number variations were comprehensively estimated by comparing them with published literature and the public databases: (1) Database of Genomic Variants (DGV) (http://dgv.tcag.ca/dgv/app/home), (2) DECIPHR (http://decipher.sanger.ac.uk/), (3) ISCA (https://www.iscaconsortium.org/), (4) ECARUCA (http://www.ecaruca.net), and (5) OMIM (http://www.ncbi.nlm.nih.gov/omim).

### 2.4. Fluorescence In Situ Hybridization (FISH)

Following the outcome of karyotype analysis and CMA results, FISH analysis was carried out to further confirm the characterization of marker chromosome. Two sets of probes were used to visualize the sSMC according to the manufacturers' instructions in this study: centromere probes specific for chromosomes X, Y (F01001, CSPX, spectrum green; CSPY, spectrum red; Beijing GP Medical Technologies, Beijing, China) and *SRY* probe (RU-LPU026, Cytocell Technologies, Cambridge, UK). The specification of *SRY* probe is as follows: red labeled *SRY* probe with two non-overlapping probes, blue labeled probe for the X centromere (DXZ1), and green labeled probe for heterochromatic region (DYZ1) in Yq12.

### 2.5. AZF Microdeletion Analysis

Microdeletions in AZF region were detected using polymerase chain reaction (PCR) technique. Specific sequence-tagged sites (STSs) were mapped in the AZF region, including SY84 and SY86 for AZFa, SY27, SY134, and SY143 for AZFb, and SY152, SY157, SY254, and SY255 for AZFc.

## 3. Results

**Figure 1:**
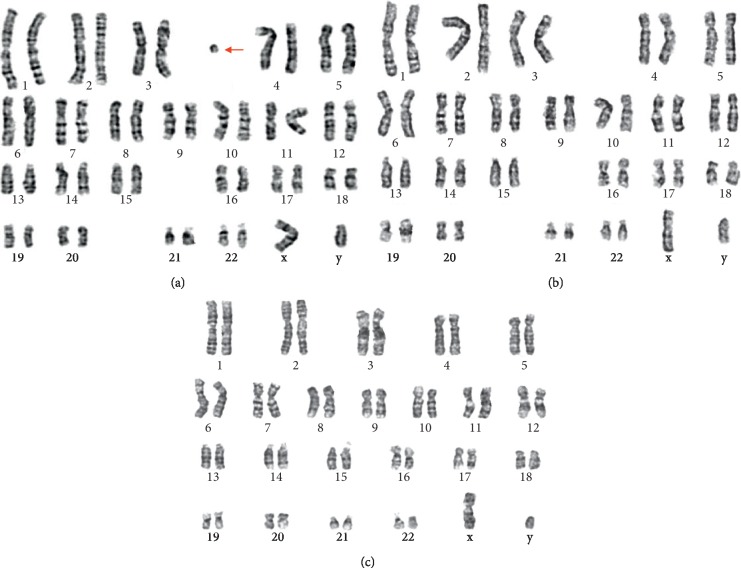
The mosaic karyotype of the patient identified by GTG banding technique with sSMC (a) and without sSMC (b). Arrow indicated the sSMC. (c) The father's karyotype: 46, XY.

**Figure 2:**
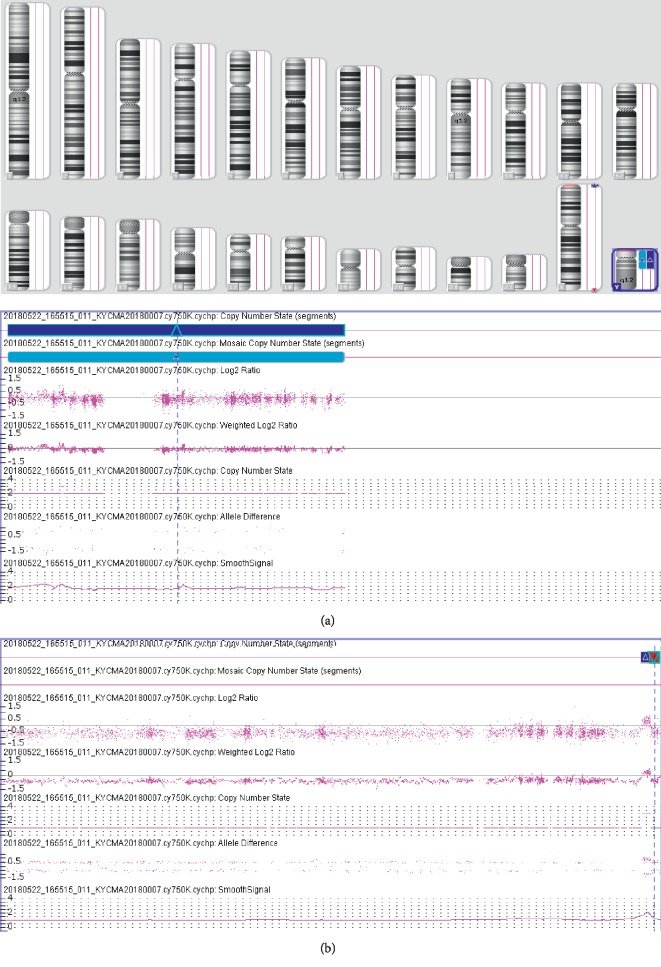
The CMA results depicted a 26.1 Mb gain of the chromosome Yp11.31q11.23 (a) and a 0.15 Mb loss of the Yq12 region (b) for the patient.

**Figure 3:**
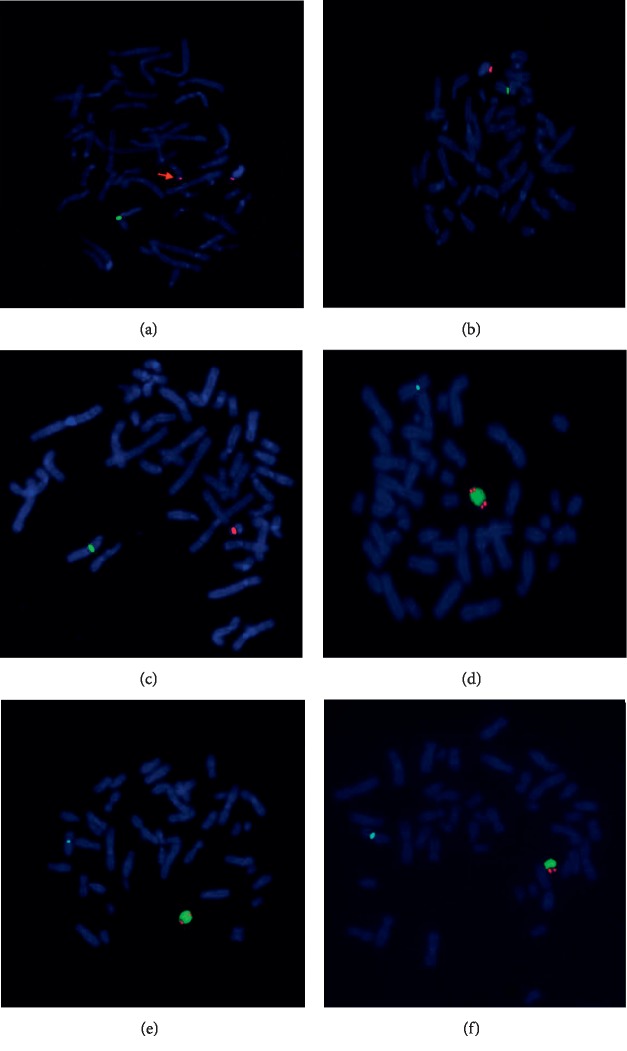
Metaphase-FISH results of two sets of probes. The centromere specific probes detected (a) two Y centromeric signals (red) in cell lines with sSMC(Y) (red arrow) and (b) one Y centromeric signal (red) in cell lines without sSMC(Y) for the patient; (c) one Y centromeric signal (red) for the father. *SRY* probe detected two *SRY* signals (red) on a same chromosome Y in cell lines with sSMC(Y) (d) and without sSMC(Y) (e) for the patient, and one *SRY* signal (red) for the father (f).

**Figure 4:**
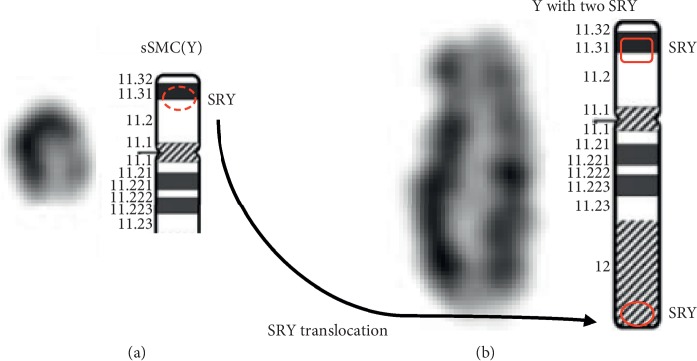
Diagram representation of SMC(Y) (a) together with chromosome Y with two *SRY* genes (b). The *SRY* of the sSMC(Y) was translocated to the apparently normal Yq terminal.

Chromosomal karyotypic analysis initially described a mosaic karyotype 47, XY, +mar[32]/46, XY[18] for the patient (Figures 1(a) and 1(b)), while his father karyotype was 46, XY (Figure 1(c)). Then, the CMA was applied to identify the sSMC for characterization. The detecting results showed arr[hg19]Yp11.31q11.23 (2, 650, 424–28, 799, 654) × 2[0.78]; arr[hg19]Yq12(59, 189, 344–59, 336, 104) × 1 (Figure 2), which illustrated that there existed a 26.1 Mb duplication of Yp11.31q11.23 and a 0.15 Mb deletion of Yq12. Subsequently, two sets of FISH probes were carried out for further verification of the sSMC on the patient and his father. FISH using centromere probes specific for chromosomes X, Y separately detected two and one chromosomal Y centromeric signals (red) in two cell lines (Figures 3(a) and 3(b)) of the patient, which catered for the karyotypic mosaicism. Nevertheless, his father carried one chromosomal Y centromeric signal (Figure 3(c)). The *SRY* probe detected two *SRY* signals (red) located in a same chromosome Y in both cell lines (Figures 3(d) and 3(e)), while his father showed a normal *SRY* signal in all cell lines (Figure 3(f)). In addition, no matter which set of probe was used, they both presented one chromosomal X centromeric signal in their respective cell lines. Based upon the fact above, we speculated that the missing *SRY* gene in the sSMC(Y) was translocated to the terminal of long arm of the “seemingly normal” chromosome Y (Figure 4). And, the sSMC(Y) was identified as *de novo* and finally described as der(Y) (pter ⟶ q11.23) (*SRY*-). In addition, no microdeletions in the AZF region were detected.

## 4. Discussion

In this study, we described a high-level mosaic sSMC(Y) male who presented severe oligospermia but no other apparent abnormalities. The G-banding analysis initially described the patient's karyotype as 47, XY, +mar[32]/46, XY[18] while subsequent CMA results further indicated that the marker chromosome could be described as sSMC(Y) with Yq12 loss. FISH further identified two *SRY* genes located on the “seemingly normal” chromosome Y while no *SRY* gene was observed on the sSMC(Y). Hence, it can be confirmed that the missing *SRY* gene in the sSMC(Y) was translocated to the distal terminal of the long arm of chromosome Y.

The sSMC can be divided into three categories based upon various shapes: (1) ring; (2) centric minute; (3) inverted duplication [4]. The phenotypes of sSMC cases were associated with a complex of factors, such as the size and origin of euchromatin, mosaicism, and uniparental disomy of sSMC's sister chromosomes [11]. Among the published infertile sSMC cases, sSMC(15) was the most frequently observed [6]. However, the genotype-phenotype correlation between sSMC and infertility is still complicated and needs to be further studied.

It is estimated that somatic mosaicism was present in 50% sSMC cases. The frequency of mosaic sSMC derived from non-acrocentric chromosome was slightly higher than acrocentric group. For rare chromosomal Y anomalies, they were more inclined to appear in the form of mosaicism with 45, X karyotype [12]. In our report, the proband showed a mosaic sSMC(Y) karyotype. To clarify the genotype-phenotype correlation of mosaic sSMC, we summarized the somatic 47, XY, +mar/46, XY males associated with spermatogenesis impairment in Table 1, according to the sSMC database [13]. Among them, three cases (Nos. 2, 6, and 7) were *de novo* and the others were not available. These sSMC cases presented varied degrees of spermatogenesis disturbance: four cases (Nos. 1, 2, 3, and 5) presented oligoasthenoteratozoospermia (OAT), and two cases (Nos. 6 and 7) presented asthenospermia. It seemed that the existence of sSMC was more frequently associated with OAT and asthenospermia rather than azoospermia in infertile male. This catered for the speculation that presence of a 47, XY, +mar/46, XY in germinal mosaicism or elimination of sSMC before or during meiosis in the germinal cells might result in low rate of spermatozoa in the sSMC carriers [14]. Moreover, it is worth mentioning that sSMC with somatic mosaicism cannot be arbitrarily screened through CMA alone, and cytogenetic analysis is still the gold standard approach in detecting these mosaic chromosomal anomalies [15].

In our study, the sSMC(Y) was finally identified as a der(Y) (pter ⟶ q11.23) (*SRY*-). The reason for his spermatogenesis impairment without apparent abnormalities was possibly due to the existence of sSMC(Y). The correlation between sSMC and infertility is complicated and under study all time. It was proposed that spermatogenic impairment due to the presence of sSMC might be responsible for oligospermia [16], for the unpaired chromatin resulting from sSMC could potentially cause meiotic arrest during spermatogenesis [17]. Meanwhile, the association between sSMC and sexual vesicle could lead to meiosis arrest and produce severe spermatogenetic impairment, which probably accounted for severe oligozoospermia and asthenozoospermia [14]. Due to the limitation of banding resolution, the karyotype of the patient's father could only be described as 46, XY. There is heterogeneity of the pericentric inversions of chromosome Y, which was the result of breakpoints in Yp and Yq [18]. If the chromosomal Y pericentric inversion in the father existed, we assumed that this might be responsible for the sSMC(Y) found in the patient, so high-resolution banding technique and FISH with Y-specific DNA probes should be carried out for further verification.

Based upon the cytogenetic analysis and CMA results, the case in our research can also be approximately viewed as 47, XY, del(Y) (q12)/46, XY. Till now, studies of male infertility on mosaic 47, XYY/46, XY are infrequent, for they could present varied degrees of spermatogenesis, ranging from normal spermatozoa to severe oligoasthenozoospermia [19, 20]. Meanwhile, Yq deletions were crucially associated with sex chromosomal mosaicism and affected the instability of chromosome Y [14]. Hence, his severe oligospermia might be due to the synthetic actions of chromosomal karyotypic mosaicism and the existence of sSMC(Y) with Yq12 deletion.

In addition, the CMA results revealed that the patient had trisomy Yp11.31q11.23 (chrY: 2,650, 424–28, 799, 654), which contains >40 genes including 3 morbidity-associated genes (*SRY*, *TBL1Y*, and *USP9Y*) with diverse clinic diseases. *SRY* (OMIM: 480000), located at Yp11.2, is responsible for initiating male sexual determination. The loss of function mutations in *SRY* can cause 46, XY sex reversal [21]. Currently, studies on *SRY* gene duplication are rare. Lehman et al. reported an azoospermic male presenting two *SRY* genes in an isodicentric Y chromosome [22]. However, whether the presence of extra *SRY* gene in patients contributed to the spermatogenic failure still needed further investigation. *TBL1Y* (OMIM: 400033), located at Yp11.2, is involved in syndromic hearing loss (Y-linked deafness-2) [23]. *USP9Y* (OMIM: 400005), located at Yq11.221, encodes a protein which functions as ubiquitin C-terminal hydrolase. The deletions of *USP9Y* have been associated with azoospermia or severe oligospermia, but it might not be essential for normal sperm production and fertility in humans [24]. In addition, the CMA also detected a monosomy Yq12 (chrY: 59, 189, 344–59, 336, 104), which contained two genes: *VAMP7* (OMIM: 300053) and *IL9R* (OMIM: 300007). Increased copy of *VAMP7* can disrupt human male urogenital development through altered estrogen action [25]. *IL9R*, including 11 exons and 10 introns, is located within the pseudoautosomal region of the Xq and Yq chromosome. It was speculated that the deletion of *IL9R* or other adjacent loci in the long-arm pseudoautosomal region might be responsible for some phenotypic features associated with Yq deletions, such as short stature, azoospermia, learning disabilities, and facial dysmorphism [26].

Although offsprings of 47, XYY/46, XY carriers suffer from risk of a hyperdiploid sex constitution [20], intracytoplasmic sperm injection (ICSI) could be adopted for infertile sSMC cases with spermatozoa [14]. Moreover, the application of preimplantation genetic diagnosis can also exert considerable influence on embryos selection to avoid unbalanced sSMC offspring [27]. However, prenatal diagnosis is still necessary regardless of what kind of choice has been made.

## 5. Conclusions

In conclusion, we identified an infertile male with *de novo* mosaic sSMC(Y) according to the G-banding, CMA and FISH analysis. The sSMC(Y) was identified as der(Y) (pter ⟶ q11.23) (*SRY*-), and its missing *SRY* gene might be translocated to the terminal of long arm in the “seemingly normal” chromosome Y. In our study, the combined application of traditional and molecular cytogenetic analysis plays a critical role in characterizing the presence, origins, and constitutions of marker chromosomes, which offers more detailed guidance and explanation in genetic counseling for the sSMC infertile carriers.

## Figures and Tables

**Table 1:** Summary of 47, XY, +mar/46, XY cases associated with spermatogenesis disturbance based upon sSMC database (http://ssmc-tl.com/sSMC.html).

No.	sSMC derived from chromosome	Chromosome karyotypic results	Parental origin	Description of the sSMC	Clinical findings
1	1	47, XY, +mar[64]/46, XY[36]	N.A.	min(1) (:p11.1 ⟶ q12:)	OAT
2	4	47, XY, +mar[46]/46, XY[54]	de novo	r(4) (::p12 ⟶ q13.1::)	OAT
3	acro-N-mar	47, XY, +mar[25%]/46, XY[75%]	N.A.	Inv dup (acro) (p10)	OAT
4	acro-N-mar	47, XY, +mar[80%]/46, XY[20%]	N.A.	Inv dup (acro) (p10)	Azoospermia
5	15	47, XY, +mar[?]/46, XY[?]	N.A.	r(15) (::p1? 2 ⟶ q11.1::)	Severe OAT
6	17	47, XY, +mar[10]/46, XY[5]	de novo	min(17) (:p11.2 ⟶ q11.1:)	Asthenospermia
7	20	47, XY, +mar[80]/46, XY[20]	de novo	mar(20)	Asthenospermia
8	X	47, XY, +mar[39%]/46, XY[61%]	N.A.	min(X) (:p11.1 ⟶ q11:)	Klinefelter-phenotype
9	Y	47, XY, +mar[32]/46, XY[18]	N.A.	der(Y) (pter ⟶ q11.23) (*SRY*-)	Severe oligospermia^*∗*^

N.A., not available; OAT, oligoasthenoteratozoospermia; ^*∗*^case of this study.

## Data Availability

The data used to support the findings of this study are included within the article.
